# Effectiveness of positive allosteric modulators of metabotropic glutamate receptor 2/3 (mGluR2/3) in animal models of schizophrenia

**DOI:** 10.1038/s41398-024-03194-2

**Published:** 2025-01-14

**Authors:** David Olivares-Berjaga, Albert Martínez-Pinteño, Natalia Rodríguez, Sergi Mas, Constanza Morén, Eduard Parellada, Patricia Gassó

**Affiliations:** 1 Department of Basic Clinical Practice, University of Barcelona, Barcelona, Spain; 2 Institut d’Investigacions Biomèdiques August Pi i Sunyer (IDIBAPS), Barcelona, Spain; 3 Centro de Investigación Biomédica en Red de Salud Mental (CIBERSAM), Barcelona, Spain; 4 Barcelona Clínic Schizophrenia Unit (BCSU), Department of Psychiatry, Institute of Neuroscience, Hospital Clínic of Barcelona, University of Barcelona, Barcelona, Spain; 5 Department of Fundamental and Clinical Nursing, Faculty of Nursing, University of Barcelona, Barcelona, Spain

**Keywords:** Clinical pharmacology, Schizophrenia

## Abstract

Schizophrenia (SZ) is a deleterious brain disorder characterised by its heterogeneity and complex symptomatology consisting of positive, negative and cognitive deficits. Current antipsychotic drugs ameliorate the positive symptomatology, but are inefficient in treating the negative symptomatology and cognitive deficits. The neurodevelopmental glutamate hypothesis of SZ has opened new avenues in the development of drugs targeting the glutamatergic system. One of these new therapies involves the positive allosteric modulators (PAMs) of metabotropic glutamate receptors, mainly types 2/3 (mGluR2/3). mGluR2/3 PAMs are selective for the receptor, present high tolerability and can modulate the activity of the receptor for long periods. There is not much research in clinical trials regarding mGluR2/3 PAMs. However, several lines of evidence from animal models have indicated the efficiency of mGluR2/3 PAMs. In this review, focusing on in vivo animal studies, we will specifically discuss the utilization of SZ animal models and the various methods employed to assess animal behaviour before summarising the evidence obtained to date in the field of mGluR2/3 PAMs. By doing so, we aim to deepen our understanding of the underlying mechanisms and the potential efficiency of mGluR2/3 PAMs in treating SZ. Overall, mGluR2/3 PAMs have demonstrated efficiency in attenuating SZ-like behavioural and molecular deficits in animal models and could be useful for the early management of the disorder or to treat specific subsets of patients.

## Introduction

Schizophrenia (SZ) is a mental disorder characterised by its chronicity, severity, and complexity [1]. The main symptoms of this disease can be divided into positive symptoms (delusions, hallucinations and disorganised speech), negative symptoms (diminished emotional expression or avolition) [1] and cognitive deficits (impairments in working memory, problem solving or social cognition) [2]. SZ affects approximately 1% of the population and mainly appears during early adulthood, affecting men slightly more than women [3].

**Fig. 1:**
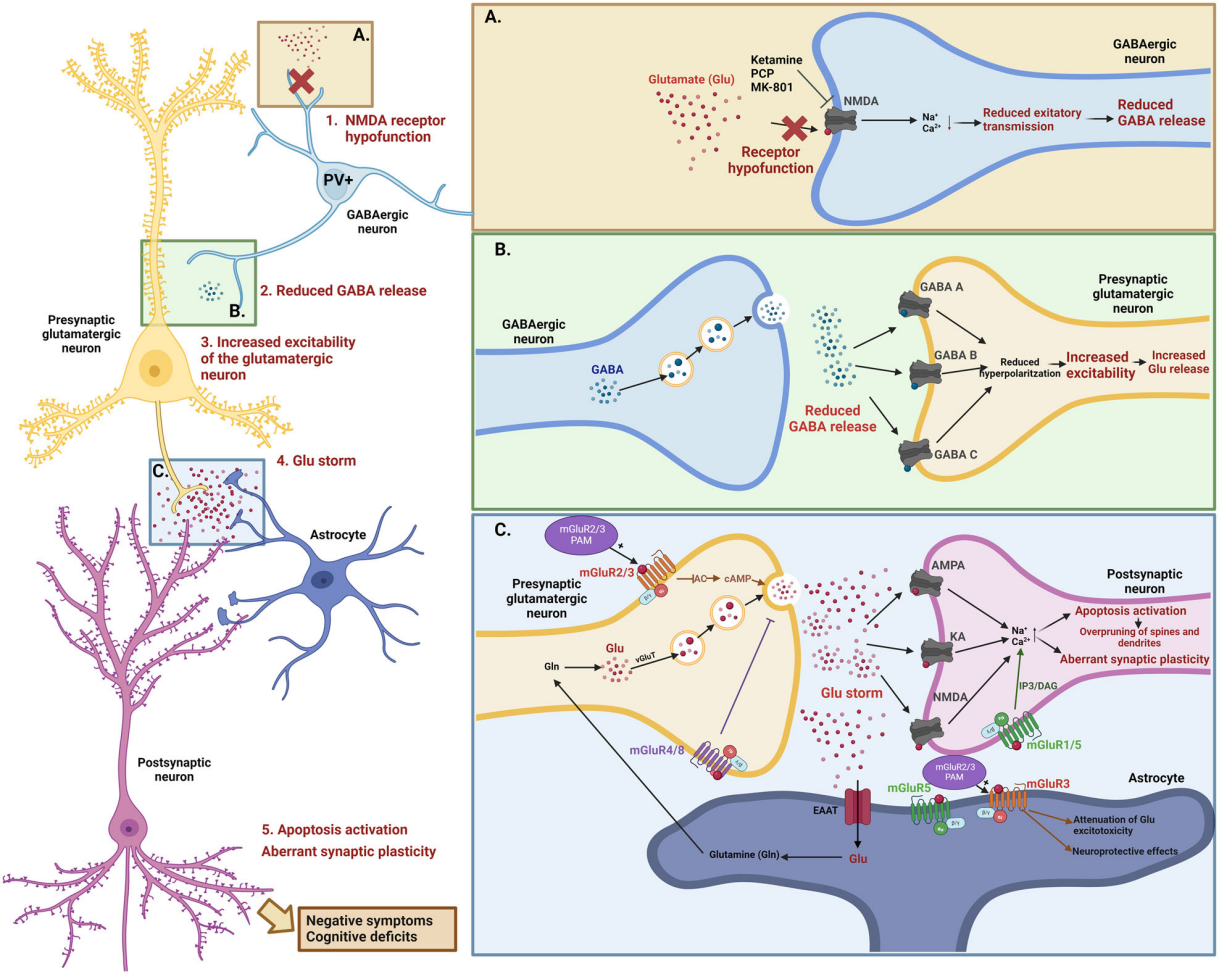
*The glutamate hypothesis of schizophrenia and modulation through the group II metabotropic glutamate receptors (mGluRs).* **A** In schizophrenia (SZ) patients, N-methyl-D-aspartate (NMDA) hypofunction in the inhibitory GABAergic neurons expressing parvalbumin (PV+) results in the reduction of excitatory transmission, attenuating GABA release. NMDA receptor antagonists, such as ketamine, phencyclidine (PCP) and dizocilpine (MK-801), promote similar molecular effects. **B** The reduced presence of GABA in the synaptic space causes the disinhibition of the presynaptic glutamatergic neurons, resulting in increased excitability and increased glutamate (Glu) release. **C** Excessive Glu, known as the Glu storm, is released from the presynaptic glutamatergic neurons and binds to the ionotropic Glu receptors, including the NMDA, α-amino-3-hydroxy-5-methyl-4-isoxazole propionic acid (AMPA) and kainate (KA) receptors, located on the postsynaptic neurons. Ionotropic Glu receptors promote excitatory synaptic transmission via the entrance of sodium (Na^+^) and calcium (Ca^2+^) ions. The excessive excitatory transmission in critical periods finally results in abnormal synaptic plasticity and the local activation of the dendritic mitochondrial apoptosis pathway and caspase-3 cascade, leading to the overpruning of spines and dendrites. The Glu released in the synaptic space is reabsorbed by the astrocytes through the excitatory amino acid transporter (EAAT). In the astrocytes, Glu is converted into glutamine (Gln) that is transported again to the presynaptic glutamatergic neurons, where Gln is reconverted into Glu and introduced into the synaptic vesicles via the vesicular glutamate transporter (VGluT). Metabotropic glutamate receptors (mGluRs) modulate the glutamatergic synapse at different points. Group I mGluRs (mGluR1 and 5) couple to the G_αs/q_ subunit of the heterotrimeric G protein, presynaptically promoting Glu release and postsynaptically potentiating the activity of the ionotropic Glu receptors that attenuate Glu-induced excitotoxicity. The presence of mGluR5 in astrocytes has been linked to neuroprotective effects. Group II mGluRs (mGluR2 and 3) couple to the G_αi/o_ subunit of the heterotrimeric G protein, presynaptically reducing Glu release. Furthermore, the presence of mGluR3 in astrocytes promotes neuroprotective effects and attenuates Glu-induced excitotoxicity. Group III mGluRs (mGluR4 and 8) couple to the G_αi/o_ subunit of the heterotrimeric G protein, presynaptically reducing Glu release. In SZ, the modulation of mGluR2/3 through positive allosteric modulators (PAMs) leads to a presynaptic reduction of Glu release, attenuating the Glu storm and promoting neuroprotective effects.

Current treatment of SZ alleviates the positive symptoms through the use of antipsychotic (AP) drugs. However, these drugs have a high percentage of non-responders (a third of patients) and are not effective in treating the negative symptoms or cognitive deficits [4, 5]. Therefore, new therapies have been proposed including those based on the neurodevelopmental glutamate (Glu) hypothesis of SZ [6]. According to this theory, N-methyl-D-aspartate (NMDA) receptor dysfunction in the inhibitory gamma-aminobutyric acid-producing (GABAergic) interneurons expressing parvalbumin (PV+) leads to the inhibition of the GABAergic system, which disinhibits glutamatergic neurons and promotes an increase in Glu release, resulting in the Glu neurotoxic storm [7]. Evidence shows that Glu neurotoxicity during the early stages of SZ can lead to excessive synaptic pruning due to increased apoptosis of the dendritic spines in critical brain areas [8] (Fig. 1).

There is much evidence pointing out alterations in glutamatergic neurotransmission associated with SZ. Postmortem brain studies have reported consistent morphological alterations in the dendrites of pyramidal glutamatergic neurons, along with a reduction in synaptic boutons. Significant findings from GWAS studies in SZ include markers associated with the glutamatergic system, including genes encoding NMDA and metabotropic Glu receptors (mGluR) *GRIN2A* and *GRM3* [9, 10]. Moreover, changes in the expression of molecules involved in the Glu pathway have also been described, including Glu receptors (NMDA, vGLUT, etc), co-agonists like D-serine, excitatory amino acid transporters (EAAT) or enzymes responsible for the neurotransmitter metabolism, such as glutamine synthetase (GS) and glutaminase (PAG) [11]. Additionally, spectroscopic magnetic resonance imaging studies have demonstrated elevated levels of Glu in treatment-naïve patients with SZ [12] and in high-risk individuals in different brain areas, including prefrontal cortex and hippocampus [13]. After psychosis onset, Glu dysfunction may also relate to the degree of antipsychotic response and clinical outcome. These findings support the development of pharmacological interventions that target the Glu system and could suggest that glutamatergic compounds may be more effective in specific patient subgroups or illness stages [11, 12].

Several new therapies targeting the glutamatergic system aim to modulate mGluR [14, 15] (Fig. 1). mGluR are members of the G-protein-coupled receptor (GPCR) superfamily, which transduces extracellular inputs into cellular signals through the activation of G-protein complexes. mGluR are classified into three groups (group I, group II and group III) according to several properties, such as the sequence homology, pharmacology, topology and biophysics between the different receptors. Novel treatments are mainly focused on mGluR group II, specifically types 2 and 3 (mGluR2/3). Presynaptic activation of mGluR2/3, which couple to the Gi_/o_ subunit of the heterotrimeric G-protein, leads to the inhibition of adenylate cyclase (AC). This inhibition, in turn, reduces cyclic adenosine monophosphate (cAMP) levels, resulting in the inhibition of calcium (Ca2+) channels and ultimately decreasing the excitability of glutamatergic cells, leading to the attenuation of Glu release [16, 17].

There have been advances in developing agonists for these receptors, such as LY354740 [18], LY379268 [19], LY404039 [20], LY2140023 [21], LY459477 [22] and LY544344 [18]. However, these drugs have not demonstrated effectiveness in the treatment of SZ in clinical trials. Consequently, a new approach involving the use of positive allosteric modulators (PAMs) has been developed. PAMs are more selective for the receptor compared to the typical agonists, present higher tolerability and can modulate the activity of the receptor for longer periods (because of less receptor desensitisation due to the lack of direct binding to the active side of the receptor). Some examples of mGluR2/3 PAMs with results in preclinical studies are LY487379 [23], JNJ-42153605 [24], JNJ-40411813 [25], JNJ-46356479 [26], AZD8529 and BINA [27]. Moreover, other molecules that such as JNJ-46281222 has been synthetized, but no preclinical research targeting SZ has been performed to date [15].

Comprehensive reviews about the role of mGluR 2/3 agonists have previously been published [28, 29], but none have focused on the effectiveness of mGluR2/3 PAMs in the treatment of SZ. Consequently, we will focus on the effects of mGluR2/3 PAMs from in vivo studies to summarise the latest findings on this topic. Most of the research has been performed in animal models and only a few studies on mGluR2/3 PAMs have been performed in clinical trials. In this review, focusing on in vivo animal studies, we will specifically discuss the utilisation of SZ animal models and the various methods employed to assess animal behaviour before summarising the findings on the effects of mGluR2/3 modulators obtained to date. By doing so, we aim to deepen our understanding of the underlying mechanisms and potential therapeutic approaches for SZ.

## Animal models of schizophrenia

Given the intricate nature of SZ and the limited accessibility to human brain samples, the utilisation of animal models has become indispensable for delving into the study of the disease and its pharmacology [30]. Currently, murine models are widely employed for this purpose. However, there is no animal model of SZ that presents the complete orchestra of SZ symptomatology. Thus, several models have to be used to reproduce the different aspects of the disease and study SZ pathophysiology and the effects of different pharmacological approaches. These preclinical models can be divided into three groups: genetic, pharmacological, and neurodevelopmental [31].

Genetic models are generated by genetic engineering, promoting mutations in genes involved in the pathophysiology of diseases and should be understood as basic scientific tools to interrogate the effects of disease associated genes [32]. These models have been widely used for elucidating the molecular basis of SZ and its symptomatology. However, the complex polygenic nature of the disease and the genetic, molecular and phenotypic overlap between psychiatric disorders complicates the interpretation and specificity of findings obtained in genetic animal models, making necessary validations in other systems to ensure their relevance to human biology [32, 33]. SZ genetic models include those with mutations in certain genes, such as *disrupted-in-schizophrenia 1 (DISC1)* [34–36]*, dystrobrevin binding protein 1* (*DTNBP1)* [37] or *neuroregulin-1 (NRG1)* [38], or those with chromosomal alterations, such as deletions in the 22q11.2 region [39]. Genetic models present different phenotypic effects in the animal depending on the gene that has been altered, resulting in deficiencies in several neuronal processes that can lead to SZ-like positive, negative or cognitive symptoms [31]. Interestingly, genetic models based on alterations in components of the glutamatergic system, such as mutant mice for subunits of the NMDA receptor (NR1, NR2A, NR2B) or for the kainate receptors, have also been developed and have shown schizophrenia-like behaviours [40].

Pharmacological models involve generating a SZ-like phenotype in the animal by an acute or chronic administration of drugs. These models are generally cheap, easy to produce and useful for studying the molecular pathways involved in the disease and the effects of drugs on reversing the deficits generated [31]. However, it is crucial to emphasise that the phenotype obtained after the drug administration might not be the same in animals that have undergone different treatments with the same drug. Characteristics such as the age of the animal, the dose of the drug or the duration of the treatment could have critical effects on the phenotype [41]. The main drugs used to induce SZ-like phenotype are dopamine enhancers and NMDA receptor antagonists. It is well known that the enhancement of the dopaminergic system is associated with the positive symptoms of SZ [42]. The most typical dopamine enhancer is amphetamine [43]. NMDA receptor antagonists promote a highly glutamatergic brain state that leads to deficiencies of other neurotransmitters, similar to what is thought to happen during the early stages of SZ. This has additionally been related to the negative symptoms of SZ and psychotic-like behaviour [28, 44]. These models with glutamatergic dysfunction are currently generated by the administration of phencyclidine (PCP) [45, 46], ketamine (KET) [47] or dizocilpine (MK-801) [46] (Fig. 1). Another pharmacological approach to generate animal models of SZ involve the modulation of the serotonergic system. Some amphetamine molecules, such as 2.5-dimethoxy-4-methylamphetamine (DOM) or 2.5-dimethoxy-4-iodoamphetamine (DOI), can activate the serotonin receptors (5-HT2A and 5-HT2C) promoting SZ-like symptoms and Glu release. Interestingly, it has been demonstrated that the simultaneous exposure to serotonergic agonists and NMDA receptor antagonists enhanced SZ symptomatology in animal models [48].

Neurodevelopmental models arise from abnormalities in neurodevelopmental processes generated from insults during gestation or the early life of the animal [31]. The neurodevelopmental processes involved in SZ pathogenesis or in the increased risk for the disorder include several non-genetic factors such as childhood trauma, drug abuse, social isolation or pregnancy and birth complications [49]. Neurodevelopmental insults determine the lifetime trajectory of the disease that can be observed in neurodevelopmental animal models as a progressive development of symptomatology [33]. These models enable the understanding of the pathology of SZ and its development. The animals present numerous deficiencies that are comparable to the positive, negative, and cognitive symptomatology of SZ. Additionally, deficiencies in the glutamatergic system have also been reported in neurodevelopmental animal models. For example, the prelimbic cortex of gestational methylazoxymethanol acetate (MAM)-exposed animals have shown epigenetic alterations in NMDAR subunits even before animal presents the emergence of dopaminergic hypersensitivity typical from this model [13]. However, these models are more expensive and complex, and eventually require surgical interventions. The main insults used to generate neurodevelopmental models of SZ are: the induction of brain lesions [50, 51]; exposure to social isolation [52], prenatal stress [53], nutritional deficits [54] or neurotoxins [55, 56]; induction of immunological responses [57]; or hypoxia [58].

Diverse approaches involving the development of neonatal pharmacological models have been proposed [44, 59–61]. These models combine a neurodevelopmental model (developed from insults during early life) with a pharmacological model (currently generated by exposing the animals to different drugs acutely or chronically). In this case, pharmacological interventions are performed during the early life stages of the animal, with the deficiencies appearing during development and maintained into adulthood. An example is the early administration of NMDA antagonists on several postnatal days (PND) that produces different symptomatology depending on the particular animal model. [44, 59–63].

It is important to emphasise that all these animal models should be understood as experimental systems to interrogate different pathways related with an interest disease rather than a direct disease model. Despite the utility and unquestioned importance of mice and other animals to investigate diseases pathological mechanisms, animals cannot be interpreted as veridical models of psychiatric disorder pathogenesis or pathophysiology and researcher had to be critical to ensure the best interpretation of results [32].

## Assessment of schizophrenia symptomatology in animal models

SZ symptoms include traits such as paranoia, disorders of perception, blunted affect and reduced cognitive capacities that are exclusive to humans or difficult to assess in animal models. However, several tests can be used to evaluate alterations in the normal behaviour of the animal that are related to positive, negative and cognitive symptoms [64, 65]. Table 1 shows a summary of the typical SZ-like behavioural assessment in animal models. Understanding the normal neurobehavior in animal models is crucial for correlating deficiencies with human symptoms. Therefore, behavioural assessments in animals must be thorough and critical to properly evaluate the schizophrenia spectrum symptoms in order to obtain reliable translational results. As stated before, as there is no animal model that can explain the entire pathophysiology of SZ by itself, there is no single behavioural test that can exactly reproduce the symptoms of patients with SZ either, and despite their usefulness, behavioural tests used to evaluate particular SZ-like symptoms have inherent limitations than can call into question their face, construct and predictive validity.

**Table 1:** Assessment of the SZ-like symptomatology in animal models of SZ.

Positive symptomatology				
Behavioural test	Measurement	Deficit in the SZ animal model	Clinical symptom	Reference
Open field test (OFT)	Travelled distance, speed, rearings and location.	Increased total distance, speed and rearing and abnormal location.	Hyperactivity	[68]
Pre-pulse inhibition (PPI)	Response to the pre-pulse.	Altered response.	Impairment of sensory motor gaiting	[70]
**Negative symptomatology**				
Forced swim test (FST)	Immobility time.	Increased immobility time.	Anhedonia and avolition	[75]
Tail suspension test (TST)	Immobility time.	Increased immobility time.	Anhedonia and avolition	[75]
Three chamber sociability test (TCST)	Social interaction time.	Reduced interaction time.	Asociality	[77]
Sucrose preference test (SPT)	Edulcorated and non-edulcorated water intake	Reduction in the edulcorated water intake.	Anhedonia, alogia and blunted affect	[78]
Elevated plus maze (EPM) test	Time expended in the open spaces.	Reduction in the time expended in the open spaces.	Alogia and blunted affect	[79]
**Cognitive symptomatology**				
Maze test variations	Time expended in known or unknown areas	Reduction in the time expended in unknown areas.	Impairment of spatial working memory	[81]
Odour span task	Odors remembered by the animal.	Reduction in the odors remembered.	Impairment of non-spatial working memory	[82]
Novel object recognition test (NORT)	Time expended exploring the known and unknown object.	Reduced time exploring the unknown object.	Impairment of visuospatial learning memory	[83]
Attentional set-shifting task (ASST)	Capacity to shift the attentions versus new stimulus.	Reduced shift capacity.	Attention and executive functioning impairment	[84]
Conditioned avoidance responding test (CART)	Capacity to avoid a negative stimulus.	Reduced capacity to avoid the stimulus.	Attention and executive functioning impairment	[85]
5-choice serial reaction time task (5-CSRTT)	Respond to the visual stimulus.	Reduced attention to the stimulus and increased response impulsivity	Attention and executive functioning impairment	[86]
Five Trial Social Memory Test (5T-SMT)	Time spent exploring the novel stimulus.	Reduced time in the novel stimulus.	Impairment in social cognition	[87]

Positive symptoms of SZ include delusions, hallucinations or disorganised speech. The complexity of such human psychotic experiences makes it difficult to find animal models to reproduce them [1]. However, patients can also exhibit psychomotor activation [66], probably caused by hyperdopaminergic activity in the brain [67]. Behavioural tests regarding positive-like symptomatology in animal models often focus on motor activity or response to stimuli [65]. The main test to evaluate hyperactivity in murine models is the open field test (OFT), which measures the distance travelled, speed and rearing of the animal and also monitors the location of the animal. Increased locomotor activity has been reported in several types of SZ animal models, including developmental, genetic and pharmacological models [65, 68]. Nevertheless, OFT as a measure of hyperactivity may not be entirely accurate as an indicator of positive symptoms. This method can overlook subtle behaviours such as sedation or stereotypy, potentially leading to incorrect conclusions about locomotor activity [69]. Another trait useful for assessing positive symptoms is sensorimotor gating that is measured with the pre-pulse inhibition (PPI) test, where a weak sensory stimulus is presented before the application of a strong stimulus. Normally, the weak stimulus (pre-pulse) is able to reduce the stimulation produced by the second stimulus, but this response is altered in animal models of SZ [70]. PPI is also considered to be affected in animals with cognitive deficits and cannot be used only to measure positive symptoms [71].

The negative symptoms of SZ include anhedonia, avolition, unsociability, alogia and blunted affect [1]. The mechanisms underlying these symptoms are not as well understood as those for positive symptoms. However, NMDA receptor dysfunction has been proposed to be involved, leading to GABAergic and dopaminergic alterations due to impaired glutamatergic signalling [72]. Negative symptoms are measured with a battery of tests that examine animal behaviour by monitoring motivation, emotional expression, social functioning and anxiety-like behaviour, among others [65]. Different behavioural tests have been used for the study of negative-like symptoms in animal models of SZ. The forced swim test (FST) [73] and the tail suspension test (TST) [74], which assess the behaviour of the animal in stressful situations, with SZ models showing increased immobility time [75], have been widely used. However, both the FST and TST are problematic due to environmental sensitivity, strain variability, potential false positives, and differing drug sensitivities, which complicates the accurate measurement of depressive-like behaviour in rodents and makes necessary to use other tests with superior validity for negative-like symptoms evaluation [76]. In the three chamber sociability test (TCST), which exposes the animal to an inanimate object and an unknown animal simultaneously, animal models of SZ show a reduction in social interaction [77]. In the sucrose preference test (SPT), which assesses the failure to feel pleasure, anxiety or depression, the animal can choose between normal or edulcorated water and the intake is measured. In this test, SZ models show a reduction in sucrose preference [78]. In the elevated plus maze (EPM), the animal is placed in a maze with open and closed arms and its tendency to explore or avoid the open spaces is monitored. SZ models demonstrate increased anxiety-like behaviour in this test, as shown by the increased time in the closed arms that is associated with a more surveillance-type behaviour [79].

Cognitive deficits in SZ affect patient memory, attention, abstract categorization, executive function, cognitive flexibility or visual processing [2].

These symptoms are possibly related to NMDAR dysfunction, which affects the GABAergic interneurons, promoting a reduction in neural γ-oscillations as proved in SZ-like animal models [80]. In animal models, cognitive function assessment is performed through the study of spatial working memory, non-spatial working memory, visual memory, problem solving and attention [65]. Working memory refers to short-term memories and the discrimination between these memories and the ones for older events. Spatial working memory is studied with different variations of the maze test (T-maze, Y-maze, Morris water maze), where the time spent by the animal in known and unknown areas of the maze is measured. SZ models show a reduction in the time spent exploring new areas [81]. Non-spatial working memory is mainly assessed with the odour span task, where animals are progressively exposed to new odors and the number of odours that an animal can remember and identify is measured. SZ models show a reduction in the number of odours that they can remember [82]. In animal models, visual memory and visual learning are related to the ability to recognise new and old objects. The main test to evaluate this is the novel object recognition test (NORT), where the animal is exposed to two different objects (known and unknown) and the time spent exploring each object is measured. SZ models show a reduced tendency to explore the new object compared to the old one [83]. The capacity for problem solving is assessed by measuring cognitive flexibility, which is usually reduced in SZ models. This can be evaluated with the attentional set-shifting task (ASST), which measures the capacity of the animal to shift attention to new stimulus through different phases [84], or the conditioned avoidance response test (CART), where the animal learns to avoid a negative stimulus such as a soft electric shock. In the CART, SZ models present deficiencies in avoiding these negative stimuli [85]. Attention and impulsivity are studied in animal models using the 5-choice serial reaction time task (5-CSRTT), which consists of exposing the animal to different visual inputs presented in different locations. The animal has to respond to this stimulus and the responses are monitored. SZ models demonstrate reduced attention and increased impulsivity [86]. Lastly, social memory, analysed as the ability to identify previously known animals. Is mainly assessed with the five-trial social memory test (5T-SMT), where the animal is first exposed to a social stimulus and then exposed to the same stimulus and a novel one, with the amount of time spent exploring the different stimuli being measured. This test is repeated multiple times to assess the short- and long-term memory of the animal. Social memory is disrupted in SZ models [87].

### Research on the use of positive allosteric modulators of mGluR2/3 in animal models of schizophrenia

mGluR2/3 PAMs act mainly as modulators of mGluR2 [15], but some PAMs, such as JNJ-46356479, also modulate mGluR3 [15]. The activation of both receptors presynaptically promote the inhibition of AC, resulting in the reduction of the Ca2+ channels activity, decreasing the neuron excitability and the Glu release and consequently reducing the hyperglutamatergic brain state. However, each receptor shows specificities, such as their localisation in the brain and distinct cell types, or even in their role [28].

mGluR2 is predominantly located in neurons present in the cerebellar granule cell layer, the media habenula, the hippocampal regions CA1 and dentate gyrus, the prefrontal cortex and the media mammillary nucleus [88]. This receptor is mostly involved in the antipsychotic effects associated with mGluR2/3 modulation, through the effects that Glu modulation have on the other neurotransmitter systems [89, 90] (Fig. 1).

mGluR3 is present in neurons and astrocytes mainly located in the thalamus, specifically in the sensory and reticular nuclei, the hippocampal CA1 region, the substantia nigra, and the piriform and entorhinal cortices [88]. This receptor has also been linked to the antipsychotic effects associated with Glu modulation in the brain, as well as with neuroprotective effects, due to its presence in astrocytes, which are thought to be involved in the attenuation of Glu excitotoxicity [90] (Fig. 1).

Overall, it has been observed that the activation of these receptors promotes antipsychotic activity and a neuroprotective effect in the brain through the modulation of the glutamatergic system, thereby becoming interesting therapeutic targets in the treatment of SZ [88–90]. These PAMs of the mGluR2/3 acting as glutamate inhibitors could be clinically beneficial during critical periods of illness, such as the prodromal stage or during the transition to psychosis, when there might be a neurotoxic Glu storm. Using these drugs during these critical stages could potentially prevent or slow the progression of the illness. Additionally, given the impact of Glu signalling in the negative and cognitive symptomatology of SZ, the use of these PAMs could be especially useful for the treatment of these symptoms [8, 91].

Summary of the following studies about the pharmacological efficacy of mGluR2/3 PAMs in animal models can be found in Table 2.

**Table 2:** Several examples of pharmacological activity of mGluR2/3 PAMs in different murine animal models.

Species	Model	Pharmacological Intervention	Behavioural effects	Molecular effect	Reference
Wistar rats	PCP 10 mg/kg (i.p) (PND 7, 9 and 11)	Acute pre-treatment LY487379 3–30 mg/kg (i.p)	↑Social discrimination	n.d	[96]
Male CD1 mice	Acute MK-801 0.3 mg/kg (i.p)	Acute LY487379 1–3 mg/kg (i.p)	↑ Novelty recognition ↑ Social interaction	n.d	[92, 97]
Male CD1 mice	Acute MK-801 0.3 mg/kg (i.p)	Acute: VU152100 0.25–0.5 mg/kg (i.p) LY487379 0.5 mg/kg (i.p)	↑ Novelty recognition ↑ Social interaction	n.d	[92]
Male CD1 mice	Chronic MK-801 0.3 mg/kg (7 days) (i.p)	Acute LY487379 1–3 mg/kg (i.p)	↑ Novelty recognition ↑ Social interaction	n.d	[92, 97]
Male CD1 mice	Chronic MK-801 0.3 mg/kg (7 days) (i.p)	Acute: VU152100 0.1–5 mg/kg (i.p) LY487379 0.1–0.25 -1 g/kg (i.p)	↑ Novelty recognition ↑ Social interaction	n.d	[92]
Male CD1 mice	Acute MK-801 0.3 mg/kg (i.p)	Persistent for 7 days: VU152100 0.5 mg/kg (i.p) LY487379 0.5 g/kg (i.p)	↑ Social interaction	n.d	[92]
Male Albino Swiss mice	Acute MK-801 0.1– 0.3 mg/kg (i.p)	Acute: VU0238429 0.25–5 mg/kg (i.p) LY487379 0.5 mg/kg (i.p)	↑ Novelty recognition Recovered PPI ↑ Working memory	n.d	[97]
Male Albino Swiss mice	Acute MK-801 0.1–0.3 mg/kg (i.p)	Acute: VU0357017 0.25–5 mg/kg (i.p) LY487379 0.5 mg/kg (i.p)	↑ Novelty recognition Recovered PPI	n.d	[97]
Male CD1 mice	Acute MK-801 0.3 mg/kg (i.p)	Acute LY487379 0.5–5 mg/kg (i.p)	↑ Acquisition and retention of the learning	↑ cGMP synthesis	[98]
Male CD1 mice	Acute MK-801 0.3 mg/kg (i.p)	Acute: VU152100 0.25–1 mg/kg (i.p) LY487379 3–5 mg/kg (i.p)	↑ Acquisition and retention of the learning	n.d	[98]
Male CD1 mice	Acute MK-801 0.3 mg/kg (i.p)	Acute: VU0238429 1–20 mg/kg (i.p) LY487379 0.5–5 mg/kg (i.p)	↑ Retention of the learning	n.d	[98]
Male CD1 mice	Acute MK-801 0.3 mg/kg (i.p)	Acute: VU0357017 0.25–1 mg/kg (i.p) LY487379 0.5–5 mg/kg (i.p)	↑ Acquisition and retention of the learning	n.d	[98]
Male Sprague-Dawley rats	Naive	Acute LY487379 (30 mg/kg) (i.p)	↑ Cognitive flexibility ↓ Impulsive-like behaviour	↑ Dopamine ↑ Norepinephrine ↑ Dopamine ↓ Glutamate	[93]
Male C57BL6J mice	Acute PCP 5 mg/kg (s.c)	Acute JNJ-42153605 (dose-dependent) (s.c)	↓ Hyperlocomotion	n.d	[99]
Male C57BL6J mice	Acute Scopolamine 0.16 mg/kg (s.c)	Acute JNJ-42153605 (dose-dependent) (s.c)	↓ Hyperlocomotion	n.d	[99]
Male C57BL6J mice	Acute Memantine 20 mg/kg (s.c)	Acute JNJ-42153605 10 mg/ kg (s.c)	n.d	↓ Brain glucose metabolism	[99]
Male Wistar rats	Naive	Acute JNJ-42153605 2.4 mg/kg (s.c)	↓ Conditioned avoidance responding	n.d	[99]
Male Wistar rats	Acute DOM (0.63 mg/kg) (s.c)	Acute JNJ-42153605 10.7 mg/kg (s.c)	↓ DOM-induced head twitches	n.d	[99]
Male Sprague- Dawley rats	Naive	Acute JNJ-42153605 20 mg/kg (p.o)	↓ Performance in rotarod test	n.d	[99]
Male C57BL6J mice	Acute PCP 2.5–10 mg/kg (s.c)	Acute JNJ-40411813 10 mg/kg (s.c)	↓ Hyperlocomotion	n.d	[99]
Male C57BL6J mice	Acute PCP 5 mg/kg (s.c)	Acute JNJ-40411813 (dose-dependent) (s.c)	↓ Hyperlocomotion	n.d	[99]
Male C57BL6J mice	Acute Scopolamine 0.16 mg/kg (s.c)	Acute JNJ-40411813 (dose-dependent) (s.c)	↓ Hyperlocomotion	n.d	[99]
Male C57BL6J mice	Acute Memantine 20 mg/kg (s.c)	Acute JNJ-40411813 10 mg/ kg (s.c)	n.d	↓ Brain glucose metabolism	[99]
Male Wistar rats	Naive	Acute JNJ-40411813 24.7 mg/kg (s.c)	↓ Conditioned avoidance responding	n.d	[99]
Male Wistar rats	Acute DOM (0.63 mg/kg) (s.c)	Acute JNJ-40411813 4.7 mg/kg (s.c)	↓ DOM-induced head twitches	n.d	[99]
Male and Female C57BL6J mice	Postnatal Ketamine 30 mg/kg (s.c) (PND 7, 9 and 11)	JNJ-46356479 during adult life (PND 80) 10 mg/kg daily for 2 weeks. (s.c)	↑ Working memory ↑ Increased social motivation ↑ Increased social memory	↑ Parvalbumin + neurons ↓ c-fos + neurons ↓ Proapoptotic protein levels	[62, 104]
Male and Female C57BL6J mice	Postnatal ketamine 30 mg/kg (s.c) (PND 7, 9 and 11)	JNJ-46356479 during adolescence (PND 35 to 60) 10 mg/kg (s.c)	↑ Working memory ↑ Increased social motivation ↑Increased social memory	↑ Parvalbumin + neurons ↑ c-fos + neurons ↓ Nitrosative Stress	[63, 105]
Murine	Undisclosed	AZD8529 (dose-dependent)	↓ Psychomotor activity ↓ Neural firing	n.d	AstraZeneca AB
Murine	PCP (undisclosed)	AZD8529 alone (57.8 to 115.7 mg/kg) or in combination with an atypical antipsychotic (5.8 mg/kg, sc)	↓ Hyperlocomotion	n.d	AstraZeneca AB
Male Sprague-Dawley rats	Acute MK-801 0.1 mg/kg (i.p)	Acute BINA 30 mg/kg (i.p)	↑ Increased social memory	n.d	[109]
Male Sprague-Dawley and Wistar rats	MK-801 0.15 mg/kg (s.c) acute	Acute BINA 100 mg/kg (i.p)	↓ Hyperlocomotion	n.d	[110]
Male Sprague-Dawley and Wistar rats	MK-801 0.5 mg/kg (s.c) twice a day for 7 days	Acute BINA 100 mg/kg (i.p)	↓ Increase in immobility time	n.d	[110]
Male C57BL6/J mice	Acute PCP 5.6–10 mg/kg (s.c- i.p)	Acute BINA pretreatment (32 mg/kg) (i.p)	↓ Hyperactivity Recovered PPI	n.d	[107]
Male C57BL6/J mice	Naive	Acute BINA pretreatment (32 mg/kg) (i.p)	↓ Stress-induced hyperthermia ↓ Anxiety-like behaviour	n.d	[107]
Male Sprague-Dawley rats	Acute PCP 5.6 mg/kg (i.p)	Acute BINA (32 mg/kg) (i.p)	↓ Hyperactivity	↓ Brain activation	[108]
Male Sprague-Dawley rats	Naïve and DOB-induced	Bath-applied (post-mortem) BINA 3 uM	n.d	↓Excitatory neurotransmission ↓ Excitatory postsynaptic currents	[111]
Male ICR mice	Acute DOB 0.3–1 mg/kg (s.c)	Acute BINA (65 mg/kg) (i.p)	↓Head twitches	↓c-fos positive cells in mPFC	[111]

*i.p* intra peritoneal, *s.c* subcutaneous, p.o *per os*, *PCP* phencyclidine, *PND* postnatal day, *DOM* dimethoxy-4-methylamphetamine, *DOB* 2.5-dimethoxy-4-bromoamphetamine, *PFC* prefrontal cortex, *PPI* pre-pulse inhibition, *n.d* not described, *cGMP* Guanosine 3,5-cyclic monophosphate.

### LY487379

N-(4-(2-methoxyphenoxy)-phenyl-N-(2,2,2-trifluoroethylsulfonyl)-pyrid-3-ylmethylamine (LY487379) was developed by Eli Lilly pharmaceuticals. In vivo studies with LY487379 [23] demonstrate its potential in reversing the pathological phenotypes in different animal models [92–97].

In naive male Sprague-Dawley rats, an acute treatment (30 mg/kg) has been shown to increase cognitive flexibility, as assessed by the ASST, suggesting that LY487378 could reverse the deficits in executive function or attention if it was administered to animal models of SZ. Additionally, the same treatment with LY487379 modifies the brain levels of Glu and other neurotransmitters (assessed through microdialysis), particularly reducing Glu levels [93].

In a neonatal Wistar rat PCP model (10 mg/kg; PND 7, 9 and 11), acute treatment with LY487379 (3–30 mg/kg) leads to the recovery in of novelty discrimination without changes in the social interaction time [96].

Furthermore, acute treatment with LY487379 (1–3 mg/kg) demonstrates a dose-dependent efficacy in reversing the deficits in novelty recognition and social interaction in an acute and chronic MK-801 model of SZ (0.3 mg/kg punctually or for 7 days, respectively) [92]. Interestingly, the simultaneous stimulation of mGluR2 and the muscarinic receptors enhances reversing of the pathological phenotypes [92, 97]. Firstly, the simultaneous administration of LY487379 and the muscarinic receptor 4 (M4) PAM VU152100 promotes the same effect as that induced by the administration of either drug alone, but these effects are promoted at subeffective doses [92]. However, continuous treatment with subeffective doses of this drug combination cannot maintain the effectiveness in reversing the deficits in novelty recognition [92]. Secondly, the simultaneous administration of LY487379 and the muscarinic receptor 5 (M5) PAM VU0238429 at subeffective doses reversed the disruptions in novelty recognition, PPI and spatial delayed alternation induced by MK-801 [97]. Lastly, the simultaneous administration of LY487379 and the muscarinic receptor 1 (M1) agonist VU0357017 at subeffective doses reverses the disruptions in novelty recognition and PPI caused by MK-801 [97]. Recently, it was demonstrated that acute treatment with LY487379 (0.5–5 mg/kg) prevented the development of cognitive deficits caused by acute MK-801 (0.3 mg/kg) exposure, with the cognitive deficits studied in the Morris water maze [98]. Again, the efficiency of LY487379 treatment was studied alongside the coactivation of the muscarinic receptors using VU0152100, VU0238429 and VU0357017, which demonstrated efficiency in ameliorating the MK-801-induced cognitive deficits in the acquisition and retention of learning, but without synergic effects [98]. Additionally, cGMP levels were studied in these animals. Increased cGMP levels were detected in the animals that had undergone cognitive training, while acute MK-801 (0.3 mg/kg) exposure significantly reduced cGMP levels and acute treatment with LY487379 (5 mg/kg) attenuated the MK-801-induced changes [98].

### JNJ-42153605

3-Cyclopropylmethyl-7-(4-phenylpiperidin-1-yl)-8-trifluoromethyl [1,2,4] triazolo[4,3-a] pyridine (JNJ-42153605) was one of the first mGluR 2/3 PAMs from Janssen Pharmaceutical. The molecule demonstrated lipophilic efficiency and in vivo efficacy in modulating the glutamatergic system during its development [24]. Acute treatment with JNJ-42153605 can, dose dependently, reduce the hyperlocomotion induced by PCP in C57BL6/J mice (acute, 5 mg/kg), while different acute doses also dose-dependently reduce the hyperlocomotion induced by scopolamine (acute, 0.16 mg/kg), an antagonist of the M1 receptor. Interestingly, these results again demonstrate an interaction between an mGluR2 PAM and a modulator of muscarinic receptors, as has been seen with LY487379. In addition, it has been observed that 10 mg/kg of JNJ-42153605 in C57Bl6/J mice reduce the hyperactivation of brain glucose metabolism induced by memantine (acute, 20 mg/kg), a non-competitive NMDA receptor antagonist [99, 100].

In Wistar rats acute treatment with JNJ-42153605 (10.7 mg/kg) reduces the conditioned avoidance response in the CART and the head twitches induced by DOM (acute 0.63 mg/kg). Moreover, JNJ-42153605 (9.4–21.5 mg/kg) dose-dependently reduces the escape response in Wiga Wistar rats [99, 101]. The drug also causes a small but significant reduction in the performance of Wistar rats in the rotarod test [99].

### JNJ-40411813

1-Butyl-3-chloro-4-(4-phenylpiperidin-1-yl)-pyridin-2(1H)-one (JNJ-40411813), a Janssen molecule also known as ADX71149 [25], demonstrates a biological effect similar to that of JNJ-42153605, but an improvement on that of the series of 1,2,4-triazolo-[4,3-a]-pyridines [99]. JNJ-40411813 presents an optimal interplay between potency, selectivity and safety [25]. Interestingly, as well as binding to mGluR2 in Wistar rats, the drug shows a moderate affinity for the 5HT2A receptor [99].

Acute JNJ-40411813 treatment (10 mg/kg) reduces the hyperlocomotion induced by exposure to PCP (acute, 2.5, 5 and 10 mg/kg), and dose-dependently decreases the hyperlocomotion induced by scopolamine (acute, 0.16 mg/kg) and PCP (acute, 5 mg/kg) in C57BL6/J mice [99]. It also reduces the hyperactivation of brain glucose induced by memantine (acute, 20 mg/kg) [99]. In Wistar rats acute treatment with JNJ-40411813 (24.7 mg/kg) reduces the conditioned avoidance response in the CART, demonstrating effects in the animal attention and executive function, while acute treatment with JNJ-40411813 (4.7 mg/kg) decreases the head twitches induced by DOM (acute, 0.63 mg/kg. Contrary to JNJ-42153605, JNJ-40411813 does not affect the performance of Wistar rats in the rotarod test, demonstrating higher safety [99].

### JNJ-46356479

8-Trifluoromethyl-3-cyclopropymethyl-7-[(4-(2-4-difluorophenyl)-1-piperazinyl) methyl]-1,2,4-triazolo[4,3-a] (JNJ-46356479) was the last drug in the series of mGluR2 PAMs developed by Janssen Pharmaceuticals. It has been used in preclinical research. The molecule belongs to the series of imidazopyridines and is characterised by a more balanced profile compared to that of the previous molecules, showing better solubility and drug-like attributes [26]. The efficacy of JNJ-46356479 in reversing neuropathological deficits and SZ-like behaviours has been studied in a postnatal ketamine mouse model (30 mg/kg; PND 7, 9 and 11) [62, 63].

Animals treated daily for 2 weeks (10 mg/kg) during adulthood (PND > 80) show a partial recovery in working memory (assessed with the Y-maze test), as well as increases in social motivation and memory (studied with the TCST and 5T-SMT). Additionally, JNJ-46356479 partially reverses the alterations in PV+ neurons and c-Fos+ cells [62]. PV+ neurons are interneurons from the GABAergic system that are affected in SZ patients [102], while c-Fos+ cells reflect neuronal activity, which is altered in SZ [103]. Last, the drug treatment modifies the apoptotic protein levels, reducing the increased apoptotic brain state present in the animal model [104].

Interestingly, chronic treatment with JNJ-46356479 during adolescence (PND 35 to 60) (10 mg/kg) reverses the cognitive and social impairments in the postnatal ketamine mouse model, while also mitigating the alterations in neuronal markers. Notably, these effects were observed in the adult mice demonstrating that the effects of early treatment with JNJ-46356479 are maintained during adulthood [63]. Additionally, the same treatment with JNJ-46356479 attenuates the imbalance in the nitrosative stress induced by an NMDA receptor antagonist [105]. Nitrosative stress is a central element in the dendritic pruning that appears in the early phases of SZ [106]. Conducting studies with varying drug treatment periods could be particularly beneficial in determining the most effective treatment window for these drugs. By examining different treatment periods in the animal model, it could simplify the translation of these drugs to different phases of the disease and treatment durations. This approach would help in identifying the optimal timing for intervention, potentially leading to significantly enhanced therapeutic outcomes.

### AZD8529

AZD8529 was an mGluR2 PAM developed by AstraZeneca AB (structure undisclosed). Not much information has been published about the efficacy of the drug in reversing the deficits present in animal models of SZ. However, the drug was used in a clinical trial involving SZ patients and AstraZeneca has demonstrated its efficacy in animal models. AZD8529 exhibits a dose-dependent capacity to reduce psychomotor activity and neural firing in rodent models. Moreover, the drug alone (57.8 to 115.7 mg/kg) or in combination with an atypical antipsychotic (5.8 mg/kg) reverses the hyper-locomotion induced by PCP (dose undisclosed) in a murine model of SZ (undisclosed) (AstraZeneca AB).

### BINA

The acute administration (30 mg/kg) of 30-[[(2-cyclopentyl-2,3-dihydro-6,7-dimethyl-1-oxo-1H-inden-5-yl)oxy]methyl]-[1,10- biphenyl]-4-carboxylic acid, better known as biphenyl-indanone A (BINA), has been shown to be effective in reversing the positive symptoms and brain activation induced by PCP (5.6–10 mg/kg) in male C57BL6J mice and Sprague-Dawley rats. Moreover, it reduces stress-induced hyperthermia, demonstrating anxiolytic activity, which has also been observed in the elevated plus maze test, where animals pre-treated with BINA spend more time in the open arms [27, 107, 108].

In animal models, acute treatment with BINA (30 mg/kg) has been observed to improve social memory in male Sprague-Dawley rats exposed to MK-801 (acute, 0.1 mg/kg) [109]. In other studies, with male Sprague-Dawley and Wistar rats, the acute administration of BINA (100 mg/kg) attenuates the increase in the immobility time in the FST after sub-chronic MK-801 exposure (twice for 7 days, 0.5 mg/kg) reduces the hyperlocomotion caused by acute MK-801 exposure (0.15 mg/kg) [110].

Furthermore, an electrophysiology study using post-mortem brain slices demonstrated that a bath-applied treatment with BINA reduced brain excitatory neurotransmission and decreased the increase in the excitatory postsynaptic currents generated by the hallucinogenic 5-HT2A receptor agonist, 2,5-dimethoxy-4-bromoamphetamine (DOB). The same study also demonstrated that acute treatment with BINA (65 mg/kg) showed the same effects in vivo, reducing the head twitches induced by DOB (0.3 mg/kg) and decreasing the number of c-Fos+ cells in the medial prefrontal cortex induced by DOB (1 mg/kg) [111].

## Drugs targeting other mGlu receptors in the treatment of schizophrenia

In addition to the modulators of mGluR2/3, the PAMs of other mGlu receptors have also shown effectiveness in reversing the pathological phenotypes in animal models of SZ.

Group I of mGlu receptors includes mGluR1 and mGluR5, which couple to the Gαs/q subunit of the heterotrimeric G protein [15]. Both receptors show antipsychotic and neuroprotective activities. The positive modulation of mGluR5 has been linked to the reversal of NMDAR hypofunction, while the activation of mGluR1 has been associated with reduced dopamine release [112] (Fig. 1).

mGluR5 modulation by PAMs produced antipsychotic effects. Firstly, CDPPB has been shown to be effective in reversing the negative and cognitive symptoms induced by MK-801 [113, 114]. Secondly, the PAM 5PAM523 reverses the amphetamine-induced hyperactivity, but its promotion of mGluR5 activity can be neurotoxic [115]. Lastly, the PAM VU0409551 enhances NMDAR function in a genetic mouse model of NMDA hypofunction rescuing the neuroplasticity deficits of the model and also effectively reversing PCP-induced cognitive impairment [116, 117]. Additionally, VU0092273 demonstrate neuroprotective effects [118].

By contrast, positive allosteric modulation of mGluR1 by VU0483605 has no neuroprotective effect [118]. However, negative allosteric modulation of mGluR1 by CFMTI reduces the hyperlocomotion induced by methamphetamine and ketamine and reverses the disruption in PPI and MK-801-induced social deficits [119]. These results demonstrate that the modulation of this receptor could be more complex than expected and follows different mechanisms to that of mGluR5.

Group III of mGlu receptors includes mGluR4/6/7/8, which couple to the G_αi/o_ subunit of the heterotrimeric G protein, producing similar molecular effects as the members of group II [15]. However, due to their location in the brain and their ability to modulate ion channels via the G_βY_ subunit, the physiological effects of this group of receptors are different to those of the group II receptors. Among the group III receptors, mGluR4 has been targeted the most by pharmacological approaches [120]. Agonists and positive allosteric modulators have been developed for mGluR4 (Fig. 1).

The mGluR4 receptor agonist LSP1-2111 can reverse the deficits generated by MK-801, but these antipsychotic effects are blocked by the antagonization of the 5-HT1A serotonin receptor, demonstrating that the effects of mGluR4 modulation depend on the serotonin system [121]. Likewise, the agonist LSP4-2022 produces antipsychotic effects alone, but these are blocked by 5-HT1A antagonism [122].

Allosteric activation of mGluR4 by the PAM ADX88178 reduces the microglia inflammatory state, which is essential for achieving neuroprotective effects, and elicits antipsychotic effects by reducing DOI-induced head twitches and MK-801-induced hyperlocomotion [123, 124]. Furthermore, the PAMs Lu AF21934 and Lu AF32615 reduce the hyperlocomotion induced by MK-801 and amphetamine, while also ameliorating the MK-801-induced alterations in the delayed alternation task and social interaction [125].

## mGluR modulation in clinical practice

As has been observed, preclinical studies in animal models demonstrate promising effects of mGluR modulation in reversing the pathological phenotype. Some drugs involving the glutamatergic system have also been studied in the clinical context, being able to cross the human blood-brain barrier and showing no serious adverse effects [126].

mGluR2/3 agonists have been used in clinical trials. It is important to mention the clinical trials involving the agonist pomaglumetad methionil (LY2140023) [127, 128]. LY2140023 is effective in reducing the ketamine-evoked BOLD signal in healthy subjects, demonstrating an in vivo effect in humans through the modulation of glutamatergic signalling [127]. It reached a phase II clinical study, where its efficacy for the treatment of SZ was compared to that of placebo and olanzapine. Unfortunately, LY2140023 was not more effective than placebo [128]. LY2979165, an mGluR2 agonist, has also been observed to reduce the ketamine-evoked BOLD signal in healthy subjects in a pharmacological magnetic resonance imaging study [127].

Regarding mGluR2/3 PAMs, a phase-I study of JNJ-40411813 showed that it was well-tolerated and reduced ketamine-induced negative symptoms in healthy subjects [129]. Additionally, JNJ-40411813 was observed to meet the secondary outcomes related to anxiety in a phase-IIa study focused on anxious depression [130]. The drug was test in patients with SZ in a phase-II study, but no results are available (ClinicalTrials.gov ID: NCT01323205).

The mGluR2 PAM AZD8529 was shown to modulate the functional response in the striatum and anterior cingulate cortex, with a greater reduction of negative symptoms in patients with higher caudate activation. Although it seemed to be effective in a patient subpopulation, it did not significantly reduce the symptomatology in the entire sample [131]. In other study, AZD8529 did not generate an effect against SZ when compared to placebo [132].

Several reasons could explain why previous SZ clinical studies with LY2140023, LY2979165, JNJ-40411813 or AZD8529 have not met the trial endpoints. For example, the inclusion of treatment-resistant patients or individuals previously exposed to medication could have masked the drug efficacy. Additionally, all studies evaluated the drug efficacy in established cases of SZ. As stated before, pharmacological interventions that target the Glu system may be more effective in specific patient subgroups or illness stages [8, 11]. Consequently, drugs targeting Glu signalling could be particularly useful during critical periods of SZ, such as the prodromal phase or the transition to psychosis, in which a neurotoxic hyperglutamatergic brain state might exist [91]. In this context, the early treatment effects of mGluR2/3 PAMs should be studied for their potential to prevent or ameliorate SZ progression. Interestingly, the comparison of JNJ-46356479 efficacy in different periods of treatment, including adulthood, adolescence or both, from adolescence to adulthood, is being evaluated in a postnatal ketamine mouse model of SZ [62, 63, 104, 105], which will help to clarify this issue. Refining the selection criteria for trial participants in future studies, considering genetic predispositions and focusing on earlier stages of the disease, could significantly enhance the outcomes for the treatment with glutamate modulators.

Data from these previous clinical studies of LY2140023 have been reanalysed to study if certain characteristics, such as the duration of the illness or the use of previous medications, are relevant for LY2140023 efficiency in treating patients [133]. Interestingly, this new post-hoc analysis revealed that a 40-mg dose in patients during the early stages of SZ who had been previously treated with D2 receptor modulators improved clinical outcomes when compared to the placebo groups. Consequently, these results demonstrate that studies involving mGlu modulators could be useful for developing personalised therapies in specific subsets of patients, such as the patients who do not respond efficiently to current treatment [133]. Additionally, these drugs could be especially useful during the early or prodromal stages of SZ [8]. Studies involving mGluR modulation should not be abandoned after a negative result since they could identify the subgroups in which these drugs could be effective.

## Conclusion

Considering all the evidence, the modulation of mGluRs is a promising target in the treatment of SZ patients who are not responding to current therapies and in the alleviation of symptoms that are currently not being efficiently treated, such as the negative and cognitive symptoms.

Overall, preclinical studies of mGluR2/3 PAMs in animal models demonstrate that they ameliorate the positive, negative and cognitive symptoms of SZ. mGluR2/3 PAMs efficiently ameliorate the positive symptoms of SZ, reducing hyperlocomotion and restoring the PPI in animal models. Moreover, they demonstrate efficiency against the negative symptoms of SZ, reversing the deficits in sociability. Lastly, the drugs effectively attenuate the deficits in cognitive flexibility and working memory in animal models. Although there are not many results from clinical trials about the effects of modulating the glutamatergic system, drugs targeting the glutamatergic system could be a useful strategy for treating specific subsets of patients or for the management of SZ during the early or prodromal phases [8, 133].

Despite the advances made to date, further research is necessary to develop effective pharmacological treatments for SZ patients and to properly alleviate negative and cognitive symptoms.
